# DESIGN, DELIVERY, AND EVALUATION OF DYADIC INTERVENTIONS WITH OLDER ADULTS

**DOI:** 10.1093/geroni/igad104.1317

**Published:** 2023-12-21

**Authors:** Karen Lyons, Kalisha Bonds Johnson

## Abstract

With increasing evidence of the interpersonal context of health and illness, there is growing interest in interventions that target the needs of the family care dyad. Yet, there are often specific challenges to the design, delivery and evaluation of dyadic interventions that are not often disseminated. This session includes four papers that will discuss the benefits (but also challenges and lessons learned) of four different dyadic interventions targeted at older adults and their care partners in both cognitive impairment and chronic illness contexts. First, Dr. Rashelle Hoffman and colleagues will present results from focus groups with rural MCI care dyads that inform the adaptation of an existing efficacious telerehabilitation physical activity intervention (TPAB). Second, Dr. Glenna Brewster and colleagues will discuss recruitment and delivery challenges, and the benefits of a pilot behavioral sleep intervention for adults with cognitive impairment and their care partners. Third, Dr. Lyndsey Miller and colleagues will report the feasibility, acceptability and preliminary efficacy of a program to support preparation for future care needs and improved well-being in care dyads living with dementia. Finally, Dr. Karen Lyons and colleagues will present findings from a pilot communication-based intervention for couples living with heart failure. The presentation will discuss the individual versus dyadic benefits of the program and challenges related to recruitment and retention. Speakers and Discussant, Dr. Kalisha Bonds Johnson, will share challenges and best-practices in designing, delivering, and evaluating interventions to optimize the health and well-being of the older adult care dyad.

